# Visuomotor Activation of Inhibition-Processing in Pediatric Obsessive Compulsive Disorder: A Magnetoencephalography Study

**DOI:** 10.3389/fpsyt.2021.632736

**Published:** 2021-04-29

**Authors:** Eman Nishat, Colleen Dockstader, Anne L. Wheeler, Thomas Tan, John A. E. Anderson, Sandra Mendlowitz, Donald J. Mabbott, Paul D. Arnold, Stephanie H. Ameis

**Affiliations:** ^1^Department of Physiology, Faculty of Medicine, University of Toronto, Toronto, ON, Canada; ^2^Neuroscience and Mental Health, The Hospital for Sick Children, Toronto, ON, Canada; ^3^Department of Human Biology, Faculty of Arts and Science, University of Toronto, Toronto, ON, Canada; ^4^Kimel Family Translational Imaging Genetics Research Laboratory, Centre for Addiction and Mental Health, Toronto, ON, Canada; ^5^Department of Psychiatry, Faculty of Medicine, University of Toronto, Toronto, ON, Canada; ^6^Department of Psychology, University of Toronto, Toronto, ON, Canada; ^7^The Mathison Centre for Mental Health Research & Education, Hotchkiss Brain Institute, University of Calgary, Calgary, AB, Canada; ^8^Cundill Centre for Child and Youth Depression, Margaret and Wallace McCain Centre for Child, Youth and Family Mental Health, Centre for Addiction and Mental Health, Toronto, ON, Canada

**Keywords:** primary motor cortex, anterior cingulate cortex, orbitofrontal cortex, response-inhibition, magnetoencephalography, pediatric, obsessive compulsive disorder, precuneus

## Abstract

**Background:** Response inhibition engages the cortico-striato-thalamo-cortical (CSTC) circuit, which has been implicated in children, and youth with obsessive compulsive disorder (OCD). This study explored whether CSTC engagement during response inhibition, measured using magnetoencephalography (MEG), differed in a sample of medication-naïve youth with OCD, compared to typically developing controls (TDC).

**Methods:** Data was analyzed in 17 medication-naïve children and youth with OCD (11.7 ± 2.2 SD years) and 13 TDC (12.6 ± 2.2 SD years). MEG was used to localize and characterize neural activity during a Go/No-Go task. Task performance on Go/No-Go conditions and regional differences in amplitude of activity during Go and No-Go condition between OCD vs. TDC were examined using two-sample *t-*tests. *Post-hoc* analysis with Bayesian *t-*tests was used to estimate the certainty of outcomes.

**Results:** No differences in Go/No-Go performance were found between OCD and TDC groups. In response to the visual cue presented during the Go condition, participants with OCD showed significantly increased amplitude of activity in the primary motor (MI) cortex compared to TDC. In addition, significantly reduced amplitude of PCu was found following successful stopping to No-Go cues in OCD vs. TDC during No-Go task performance. Bayesian *t-*tests indicated high probability and large effect sizes for the differences in MI and PCu amplitude found between groups.

**Conclusion:** Our preliminary study in a small medication-naïve sample extends previous work indicating intact response inhibition in pediatric OCD. While altered neural response in the current study was found during response inhibition performance in OCD, differences localized to regions outside of the CSTC. Our findings suggest that additional imaging research in medication-naïve samples is needed to clarify regional differences associated with OCD vs. influenced by medication effects, and suggest that MEG may be sensitive to detecting such differences.

## Introduction

Obsessive compulsive disorder (OCD) is characterized by recurrent, intrusive thoughts and/or repetitive ritualistic behaviors (1). These symptoms are associated with significant distress, and deficits in occupational, academic, and social functioning (2). The lifetime prevalence of the disorder is 2.3% in the general population and it affects up to 2% of children and youth (3, 4). The average age of onset for pediatric OCD is 11 years of age (5–7). Earlier symptom onset has been associated with increased illness severity and persistence (8). A focus on understanding brain processes linked to OCD in childhood and adolescence presents the opportunity to measure neural changes that may be directly associated with the disorder, rather than with potential confounds such as long-term medication exposure, illness duration, or learned strategies for behavioral compensation (9).

Response-inhibition is defined as top-down inhibitory control aimed at suppressing responses to external or internal stimuli (10). Response inhibition as a cognitive process is postulated to be linked to impaired control over obsessive and compulsive symptoms in OCD (11, 12). Although previous meta-analytic data have found evidence of response inhibition impairments in adults with OCD (13), a recent meta-analysis indicated that the effect size for differences in response inhibition performance between children and youth with OCD vs. controls is approaching zero (12), suggesting that impaired laboratory-based task performance in this domain may not be a feature of pediatric OCD, or may develop over time when illness symptoms persist. Nonetheless, the study of response inhibition task performance in pediatric OCD may provide key insights into the presence of alterations in inhibitory response circuitry which is thought to drive illness symptoms in everyday settings (6, 11, 14, 15).

The cortico-striato-thalamo-cortical (CSTC) circuit, comprised of the supplementary motor area (SMA), inferior frontal gyrus (IFG), orbitofrontal cortex (OFC), projections from the anterior cingulate cortex (ACC), white matter tracts including the anterior corpus callosum, cingulum bundle, and anterior limb of the internal capsule and subcortical regions, such as the striatum, subthalamic nucleus, and thalamus are implicated in habitual control and response inhibition (16). Excessive CSTC activity has been postulated to disrupt attention shifting processes and contribute to increased directed attention to threat and to neutralizing threat, expressed as obsessions and compulsions, respectively, in OCD (6). Among CSTC circuit regions, fronto-cortical regions, such as the ACC, OFC and IFG, may be particularly relevant in OCD based on the role of the ACC in supporting cognitive control and error-monitoring, the role of the OFC in emotional control, such as selective judgement or weighing of consequences (17, 18), and the IFG in action inhibition (19).

A meta-analysis of task-related functional magnetic resonance imaging (fMRI) studies (*n* = 2,345, mean age = 31.9 years) suggested that response inhibition elicits altered task-evoked brain response in adults with OCD compared to controls, including greater ACC activation (20). A limited number of neuroimaging studies have examined links between brain structure or function and response inhibition in pediatric samples (21). Available fMRI studies exploring response inhibition and related cognitive tasks have found both increased and reduced activation in OFC, medial prefrontal cortex, ACC, motor regions, and caudate nucleus during task performance in children and youth with OCD vs. TDC (22–25). A recent EEG study found hyperactivity within the right IFG in pediatric OCD that corresponded with improved response inhibition performance in OCD vs. controls, a finding that was interpreted to signal pathological CSTC hyperactivity (14). One important limitation of the available literature exploring neural response to response inhibition is that studies have thus far largely included both medicated and un-medicated children and youth with OCD. However, medication exposure may be an important confound given recent findings that selective serotonin reuptake inhibitors (SSRIs), commonly prescribed for treatment of OCD, have effects on brain structure, function, and neurochemistry (26, 27), including within the CSTC circuitry (26). SSRIs have also been associated with brain changes in children undertaking medication treatment targeting anxiety symptoms (28). Such findings have resulted in calls for increased research in medication naïve samples to disentangle neural response patterns that may be linked to medication effects rather than illness etiology (26).

While most neuroimaging studies in children and youth with OCD have focused on structural differences or have characterized neural activity in OCD using fMRI or EEG (11, 14, 23, 27), few studies have used magnetoencephalography (MEG) to study neural response (29–31), an imaging technique with some key advantages. Compared to fMRI, MEG offers higher temporal resolution due to minimal distortions from muscle artifacts (32), and provides a more direct measure of electrical activity of neurons as opposed to the hemodynamic response measured by fMRI, also enabling measurement of event-related frequencies (33, 34). As MEG records neuromagnetic activity with highly sensitive magnetic sensors, it also allows for tracking of neural activation with less signal interference and higher spatial resolution compared to EEG (35). Thus far, very few studies have used MEG to study neural response in OCD. We are aware of just three MEG studies in children and youth (29–31). One of these three MEG studies examined neural response during a cognitive flexibility task and found increased amplitude of prefrontal cortex response in participants with a primary clinical diagnosis of OCD vs. those with autism spectrum disorder or attention deficit hyperactivity disorder (*n* = 88, ages 8–15 years) (31).

The current study is the first, to our knowledge, to use MEG to examine whether CSTC alterations are present during inhibitory control performance in a medication-naïve sample of children and youth with OCD. Based on prior literature, it was hypothesized that children and youth with OCD would feature increased amplitude of neural response within frontal CSTC regions during response inhibition performance compared to TDC.

## Materials and Methods

### Participants

All participants were recruited from The Hospital for Sick Children in Toronto. Informed consent was obtained from all parents and assent from all participants in accordance with the Declaration of Helsinki, and the study was approved by the Hospital Research Ethics Board. Participants received a picture of their brain and were compensated for parking costs during study visits. Structural MRI, MEG at rest and during Go/No Go Task performance, and behavioral data were acquired in 20 medication-naive children and youth (ages 8–16 years, mean age = 11.9 years ± 2.1 SD, 11M/9F), diagnosed with OCD by a child psychiatrist (PDA, SHA) or clinical psychologist (SM) in accordance with the Diagnostic and Statistical Manual of Mental Disorders (DSM) criteria, and 14 TDC (ages 8–16 years, mean age = 12.3 years ± 2.1 SD, 7M/7F). All participants were right-handed. Exclusion criteria consisted of prior psychopharmacological treatment exposure, a history of chronic neurological disorders, any previous serious head injury resulting in loss of consciousness, history of bipolar disorder, psychosis, or schizophrenia spectrum disorder in participants with OCD, or any history of psychiatric disorder or psychiatric diagnoses among family members within the immediate family based on parent report in TDC.

### Clinical Characterization in OCD

The diagnosis of OCD was confirmed using the Schedule for Affective Disorders and Schizophrenia for School-Age Children-Present and Lifetime version (K-SADS-PL) (36). The severity of obsessive-compulsive symptoms in children and youth with OCD was assessed using the Children's Yale-Brown Obsessive-Compulsive Scale (CY-BOCS) and the Toronto Obsessive-Compulsive Scale (TOCS) (37). The CY-BOCS is a 10-item, clinician-rated instrument with items 1–5 assessing the severity of obsession symptoms and items 6–10 assessing the severity of compulsion symptoms, the sum of which make up the total score (total score <5 = transient, 5–13 = mild, 14–24 = moderate, 25–30 = moderate-severe, >30 = severe OCD symptoms) (38). The TOCS is a 21-item parent or self-report questionnaire, adapted from the CY-BOCS, providing additional quantitative information on obsessive-compulsive traits in children and youth (37).

### Magnetic Resonance Imaging

MRI scans were obtained for all participants on a 3.0T Siemens TIM Trio scanner, T1-weighted images were acquired using a 3D MPRAGE Grappa 2 protocol (TR/TE = 2,300 per 2.96 ms, voxel size 1.0 × 1.0 × 1.0 mm). During the scan, children were positioned to watch videos via goggles to minimize head motion. Fiducials placed prior to MEG scanning were kept in place during the MRI for later co-registration.

### Magnetoencephalography Recordings

A whole-head 151 channel CTF MEG system placed in a magnetically shielded room was used to record neuromagnetic activity. MEG data were collected continuously at a rate of 1,200 samples per second and were filtered at 0.3–300 Hz. Before data acquisition, each participant was fitted with one fiducial localization coil placed at the nasion and two placed at the preauricular points to localize the position of the head relative to the MEG sensors. Participants lay supine with their eyes open and fixated on a black cross (2 × 2 cm) on a semi-transparent screen placed 50 cm from their eyes. To monitor eyeblinks and saccades, electrooculograms were placed distal to the lateral canthus of each eye, and one on the left mastoid process. To monitor head movement, a head-tracking system was used during data acquisition.

### Go/No-Go Task

The Go/No-Go task is a cognitive task consisting of two conditions: a Go and a No-Go condition. The Go condition serves as a control (visuomotor engagement) condition. In this condition, only a Go cue (green cross) is presented. The No-Go condition serves as the inhibitory control condition. In this condition, both a Go cue (green cross, cue to respond quickly) and No-Go cue (red cross, cue to withhold response) are presented, measuring the ability to withhold a response (39). The No-Go condition is considered to have a higher cognitive load than the Go condition due to the need to discriminate between stimuli and cognitively select for an appropriate response (39, 40).

#### Go Condition

As described in detail by Dockstader et al. (40), during the Go condition (serving as a control condition), participant's eyes were open and fixated on a black cross. Their dominant hand was resting at their side with their thumb resting on a button box response button. The black cross presented on the screen was pseudo-randomly replaced with a green cross, temporally jittered between 1.5 and 2.5 s, and accompanied by a static visual contrast grating in the lower visual field. The location and dimensions of this contrast grating have been shown to elicit a strong visual evoked field ~75 ms after cue onset (41). During Go trials, participants were instructed to press the button with their dominant thumb immediately following the presentation of the green cross. The timing of the button-press in response to the green cross was recorded as the Go reaction time. Each participant underwent 100 Go task trials (40).

#### No-Go Condition

During the No-Go condition, the black fixation cross was replaced with either a green or red cross accompanied by a static visual contrast grating. Participants were instructed to press the button immediately following the presentation of the green cross and were instructed to *inhibit* a button-press (withhold response) following the presentation of the red cross. No-Go reaction time was recorded as the timing of button-press in response to the green cross during this condition. Stop errors were recorded as any button-press following a red cross (i.e., error presses). There were 197 green crosses and 100 red crosses presented, pseudo-randomly, such that 67% of trials were green crosses (Go cues) and 33% were red crosses (No-Go cues). The ratio of Go to No-Go stimuli was selected so that the majority of trials were Go trials (requiring a button-press). This required participants to inhibit the tendency to respond during the No-Go trials, in keeping with previous use of this task during MEG recording in pediatric samples (40–43). The presentation order was counterbalanced across all participants in both groups.

### MEG Data Pre-processing

Data quality control was implemented including visual inspection of the time points of overt saccades or eye blinks and exclusion of any trials including eye blinks prior to cue onset. Any trial where head motion was ≥5 mm was excluded, consistent with previous pediatric MEG studies (40, 44, 45). MEG datasets for all participants were processed and analyzed with Brainstorm (46), which is freely available for download under the GNU general public license (http://neuroimage.usc.edu/brainstorm). Data were down sampled to 600 samples per second prior to processing. For both Go and No-Go conditions, datasets corresponding to the timing of visual cue were defined with an epoch time of −400 ms to 1,000 ms, as a visual response is expected to occur within this time range. DC offset, which is signal noise that occurs at cue onset, was removed and a baseline epoch was defined as −100 ms to 0 ms. The datasets corresponding to time of expected button-press response (i.e., button presses to Go cues and error presses to No-Go cues) were defined with an epoch time of −300 ms to 300 ms, around the motor response. This parameter allowed for analysis of any neural activity occurring prior to or following the button-press. DC offset was removed using the baseline computed for each output file and bandpass filtered from 1 to 40 Hz, with a Notch filter of 60 Hz (46). MEG data was co-registered with MRI data to create high resolution, three-dimensional, differential images of neural activations around the time of motor responses to Go and No-Go trials, relative to baseline.

### MEG Analysis

#### Source Localization and Extraction of Response Amplitude Data

Latency of visual response in the average group waveform activity across sensors in both OCD and TDC groups in Go and No-Go conditions was first identified. The time of peak activity, at ~100 ms (expected time of visual response), was recorded as the latency of visual response to the visual cue in each group. We identified maximum amplitude of activation at peak timepoints in the group average waveforms for each group (OCD and TDC) around the time of motor responses for Go button presses and No-Go stop errors (error presses). We identified maximum amplitude of activation for successful stops to No-Go cues at peak timepoints corresponding with the time of No-Go cue onset in the group average waveforms for each group (OCD and TDC). An average activation map for each group (OCD and TDC) was created for Go button presses, No-Go stop errors, and No-Go successful stops by subtracting the average of noise data during the baseline epoch (−100 ms to 0 ms) from the active epoch (0 ms to 300 ms), with a linear minimum norm estimation algorithm applied (46, 47). Average activation maps for each group (OCD and TDC) were used to visualize and identify any regions of activation corresponding with peak time points for motor responses (derived from the group waveform) during both Go button presses and No-Go stop errors, and for regions of activation corresponding with successful stops during No-Go cue onset). Subsequently, response amplitudes and standard deviation were extracted at the participant level based on group level peak activations between 100 and 300 ms, as peak activations for group waveforms were within this time window (for Go button-press, No-Go stop errors, and No-Go successful stops). We confirmed regions of activations for each individual were the same as the regions activated at the average group level by creating activation maps corresponding with peak time points for each individual participant.

#### Statistical Analyses

Two-sample *t*-tests were used to examine between-group differences in Go (amplitude of activation and reaction time to button presses) and No-Go task performance (amplitude of activation during stop errors and number of error presses). Given the small sample included in this MEG analysis, uncorrected *p* < 0.05 was used to identify regions that differed between groups or approached significant differences.

The Multivariate Imputation by Chained Equations (MICE) package on R was used to interpolate random missing values due to missing markers indicating time of cue presentation in dataset files or missing clinical scores (48). Statistical outliers in amplitude of activation were removed using the Box Plot Statistics function in the R Graphics Devices (grDevices) package from calculation of correlation analyses (48).

#### Exploratory *Post-hoc* Analyses

##### *Post-hoc* Bayesian Analyses

We used The Bayesian First Aid R package to calculate Bayesian *t-*tests (48), enabling estimation of the precision of the effects of between-group regional differences found in the present study. The Bayesian framework was used here to reallocate credibility across the range of candidate parameter values based on the means and standard deviations found in our study data (49). The Bayesian approach provides credibility information by defining the *posterior distribution* (the probability of an observed effect), derived from Markov Chain Monte Carlo (MCMC) sampling probabilities (50). MCMC is an iterative process which generates random samples from a set of parameter values to create a distribution from which we can determine the likelihood or probabilities of the observed data (51). A Bayesian *t-*test indicates the probability of a difference in means between groups and provides effect size estimates (computed as Hedge's *g*) for the between-group difference examined. In addition, the 95% high density interval (HDI) is provided, indicating the range within which 95% of the most credible values for the effect size fall.

##### Time-Frequency Response

In order to explore the frequencies driving the responses during the Go and No-Go task conditions, time-frequency response (TFR) plots were generated for regions that differed between groups during source localization. A Morlet wave analysis of changes in oscillatory frequency was used by selecting for virtual sensors associated with regions of interest. A reduction in frequency oscillations was specified as event-related desynchrony (ERD), whereas an increase in frequency oscillations was specified as event-related synchrony (ERS). Peak frequency oscillations were identified in delta (0.5–3 Hz), theta (4–7 Hz), alpha (8–12 Hz), beta (13–29 Hz), and gamma (30+ Hz) frequency bands. Time-frequency activity was analyzed relative to baseline activity (activity prior to cue onset at 0 ms).

##### Correlation of Event-Related Amplitude With Clinical Scores

Exploratory correlation analysis was conducted to examine whether regions that differed between groups during response inhibition were associated with clinical symptoms. Correlations were computed using Pearson correlation on R Statistical Analysis (48).

## Results

### Participants

The demographic characteristics of the total sample by group are presented in Table 1. Based on the CY-BOCS, average symptom severity in participants with OCD was in the high moderate range (22.84 ± 4.5 SD). Data was adequate across participants with respect to head motion and apparent perception of cue (i.e., no participant scan from each trial included head movement ≥ 5 mm, or overt saccades or eye movements occurring between −200 and 0 ms prior to cue onset). The initial sample included 20 OCD participants and 14 TDC participants. Two OCD participants were removed due to missing MRI scans, and one additional OCD participant was removed due to missing button-press markers in MEG data files and no available datapoints to perform imputation. Similarly, one TDC participant was removed due to missing MEG date files (button-press markers). For behavioral task performance of button presses, the total number of participants analyzed in the OCD group was *n* = 17 in both the Go condition and No-Go condition. The total number of TDC participants included in behavioral task performance analysis was *n* = 13. Regional activation analyses were carried out in *n* = 17 OCD and *n* = 13 TDC participants in the Go condition, and *n* = 14 OCD and *n* = 13 TDC participants in the No-Go condition. Of note, for the regional brain activation analyses, three OCD participants (of the total available 17) were excluded in the No-Go condition analysis of stop errors due to the absence of error presses. Three other OCD participants (of the total available 17) were excluded in the No-Go condition analysis of successful stopping due to the absence of No-Go cue markers corresponding to presentation of No-Go cue.

**Table 1:** Overall sample demographic characteristics.

	**TDC (Mean ± SD)**	**OCD (Mean ± SD)**
Number of Participants (Male/Female)	13 (6/7)	17 (10/7)
Age	12.6 ± 2.2 years	11.7 ± 2.2 years
Medication Status	N/A	Medication-naive
CY-BOCS Total Score	N/A	22.84 ± 4.5
CY-BOCS Compulsion Subtotal	N/A	11.82 ± 2.7
CY-BOCS Obsession Subtotal	N/A	10.93 ± 2.3
TOCS Total Score	N/A	13.81 ± 21.9

*TDC, typically developing controls; OCD, obsessive compulsive disorder; SD, standard deviation; CY-BOCS, Children's Yale-Brown Obsessive-Compulsive Score; TOCS, Toronto Obsessive Compulsive Score*.

### Go/No-Go Task Performance

There were no significant between-group differences in button press reaction time to the Go visual cue during the Go condition (t = −1.48, df = 28, *p* = 0.15), nor were there differences in button press reaction time to Go cues during the No-Go condition (t = −1.57, df = 28, *p* = 0.13). We also found no significant between-group differences in the total number of stop errors made following No-Go visual cue presentation (t = −0.25, df = 28, *p* = 0.80) (see Table 2 for details).

**Table 2:** Behavioral performance across reaction time.

	**TDC ms (±SD)**	**OCD ms (±SD)**	***p***
Go: Mean Reaction Time	321 (± 62.9)	390 (± 158.4)	0.15
No-Go: Mean Reaction Time	423 (± 81.3)	485 (± 122.5)	0.13
Number of Errors	10.69 (± 10.68)	11.76 (± 12.27)	0.80

*Mean reaction time, in milliseconds (ms), of button-press in response to Go visual cue in Go condition and incorrect button-presses in response to stop visual cue in No-Go condition. Number of errors in youth with OCD and TDC during No-Go trials. p-values represent significance of reaction times between-groups. TDC, typically developing controls; OCD, obsessive compulsive disorder; SD, standard deviation*.

### Regional Activation During Task Performance

#### Source Localization and Amplitude in the Go Condition

**Figure 1:**
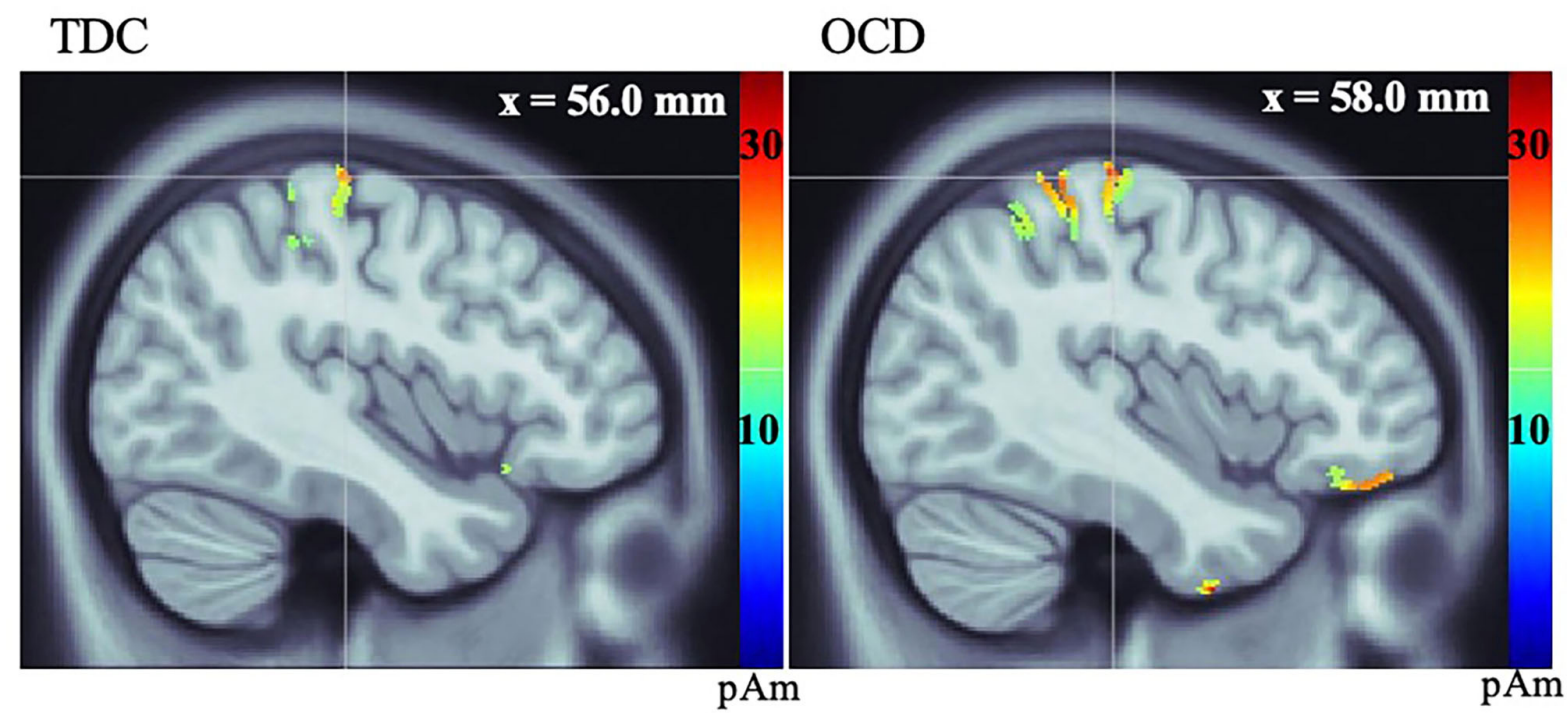
Source localization, measured in picoamperes (pAm), of group average response of primary motor cortex (MI) in TDC (left), and OCD (right) groups during button-press to Go visual cue.

Both groups showed activation in the primary visual cortex (V1) following presentation of the Go visual cue and the contralateral primary motor cortex (MI) following button-presses to the Go visual cue. There were no significant between-group differences in the latency or amplitude of V1 activity (t = 0.86, df = 28, *p* = 0.40) (Supplementary Figures 1, 2). We found that the average amplitude of MI activity at the time of the button-press was increased in the OCD vs. TDC group (t = −2.35, df = 28, *p* = 0.03) (see Figure 1, Table 3).

**Table 3:** Latency and amplitude of activation during go and no-go conditions.

		**Controls**		**OCD**		***p***
		**Latency ms (±SD)**	**Amplitude pAm (±SD)**	**Latency ms (±SD)**	**Amplitude pAm (±SD)**	
Go	V1	94.5 (± 11.1)	64.3 (± 27.8)	100.3 (± 18.5)	55.9 (± 25.6)	0.40
	MI	N/A	26.5 (± 14.2)	N/A	46.9 (± 28.6)	0.03^*^
No-Go	PCu	164.0 (± 8.7)	46.9 (± 22.2)	97.4 (± 10.2)	23.7 (± 8.7)	0.001^*^
	OFC	179.0 (± 9.1)	31.7 (± 24.1)	148.0 (± 6.43)	21.3 (± 15.6)	0.19
	ACC	178.0 (± 8.2)	27.4 (± 32.0)	141.7 (± 9.9)	23.5 (± 10.4)	0.72

*Latency, measured in milliseconds (ms), and amplitude, measured in picoamperes (pAm), values for regions most active at the group level in the Go and No-Go conditions. Uncorrected p-values represent significance of amplitudes between-groups. OCD, obsessive compulsive disorder; V1, primary visual cortex; MI, primary motor cortex; PCu, precuneus; OFC, orbitofrontal cortex; ACC, anterior cingulate cortex; SD, standard deviation*.

* *p < 0.05 (uncorrected) for amplitude of activation*.

#### Source Localization and Amplitude in the No-Go Condition

**Figure 2:**
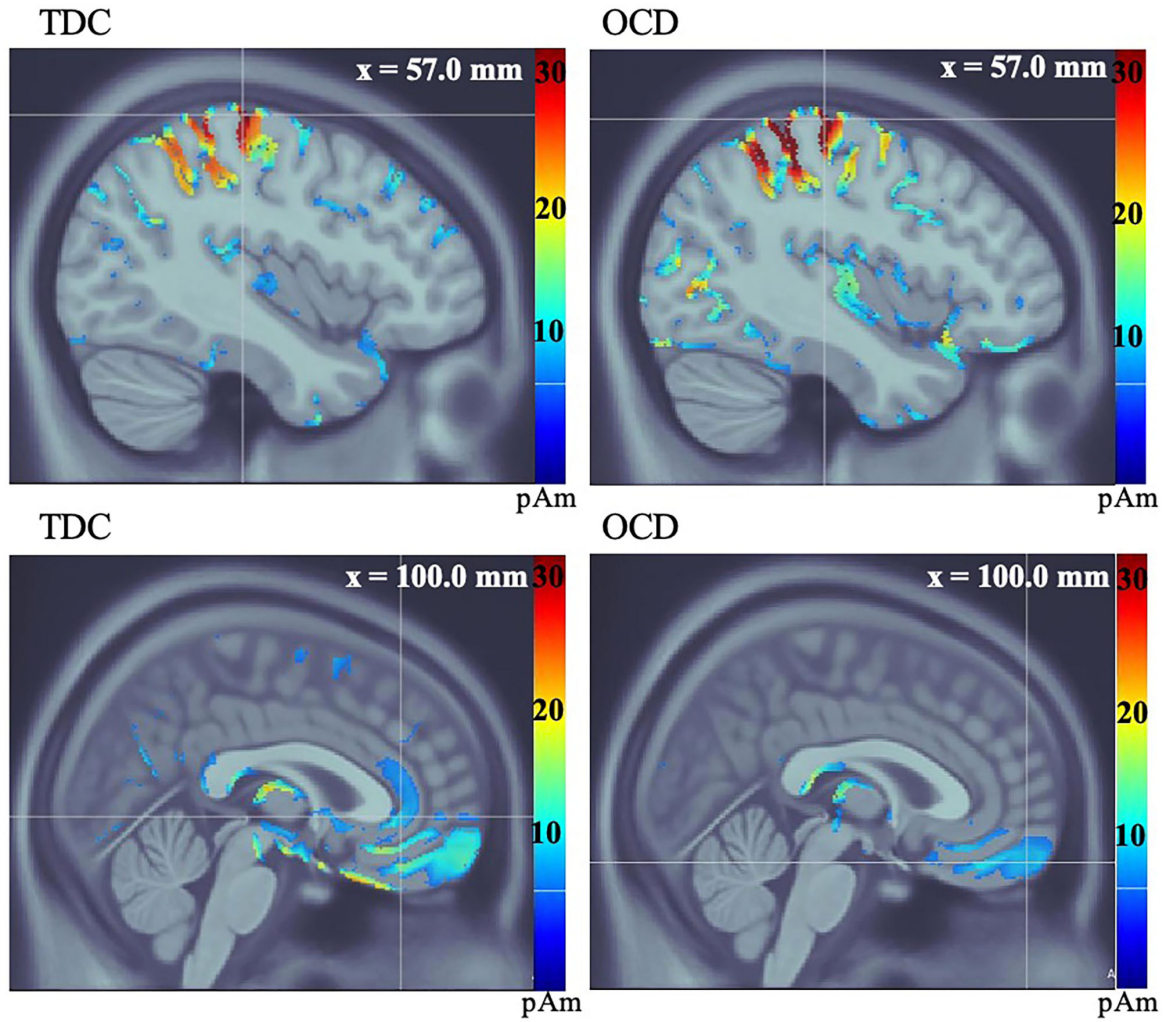
Average group source localization during incorrect button-press in response to stop visual cue in No-Go condition, measured in picoamperes (pAm). TDC group motor (MI) response (top left), and orbitofrontal, and anterior cingulate cortices (bottom left). OCD group motor (MI) response (top right), and orbitofrontal cortex (bottom right). The OCD group does not show activation of the ACC at the group level (bottom right).

Both groups showed activation in V1 and precuneus (PCu) during successful stopping to the No-Go cue. No significant between-group differences in latency or amplitude of V1 activation were found. There was a significant between-group difference in amplitude of activation in the PCu during successful stopping to the No-Go cue, such that participants with OCD featured reduced amplitude at the group level, compared to TDC (t = 3.71, df = 25, *p* = 0.001) (Supplementary Figures 3, 4). In both groups, MI was active at the time of stop errors and the OFC was active after stop errors. There were no significant differences in MI. Although the OFC mean amplitude was lower in OCD, group means were not significantly different on group-wise comparisons (t = 1.34, df = 25, *p* = 0.19). Average group activity of the ACC was observed in the TDC group after stop errors. On further examination, ACC activity was found following stop errors in 10 of 14 OCD participants (based on participant-level activations). No significant between-group difference in ACC amplitude was found (t = 0.36, df = 21, *p* = 0.72) (see Figure 2, Table 3).

### *Post-hoc* Exploratory Analyses

#### Bayesian Analyses of Between-Group Differences in Regional Brain Activation

**Figure 3:**
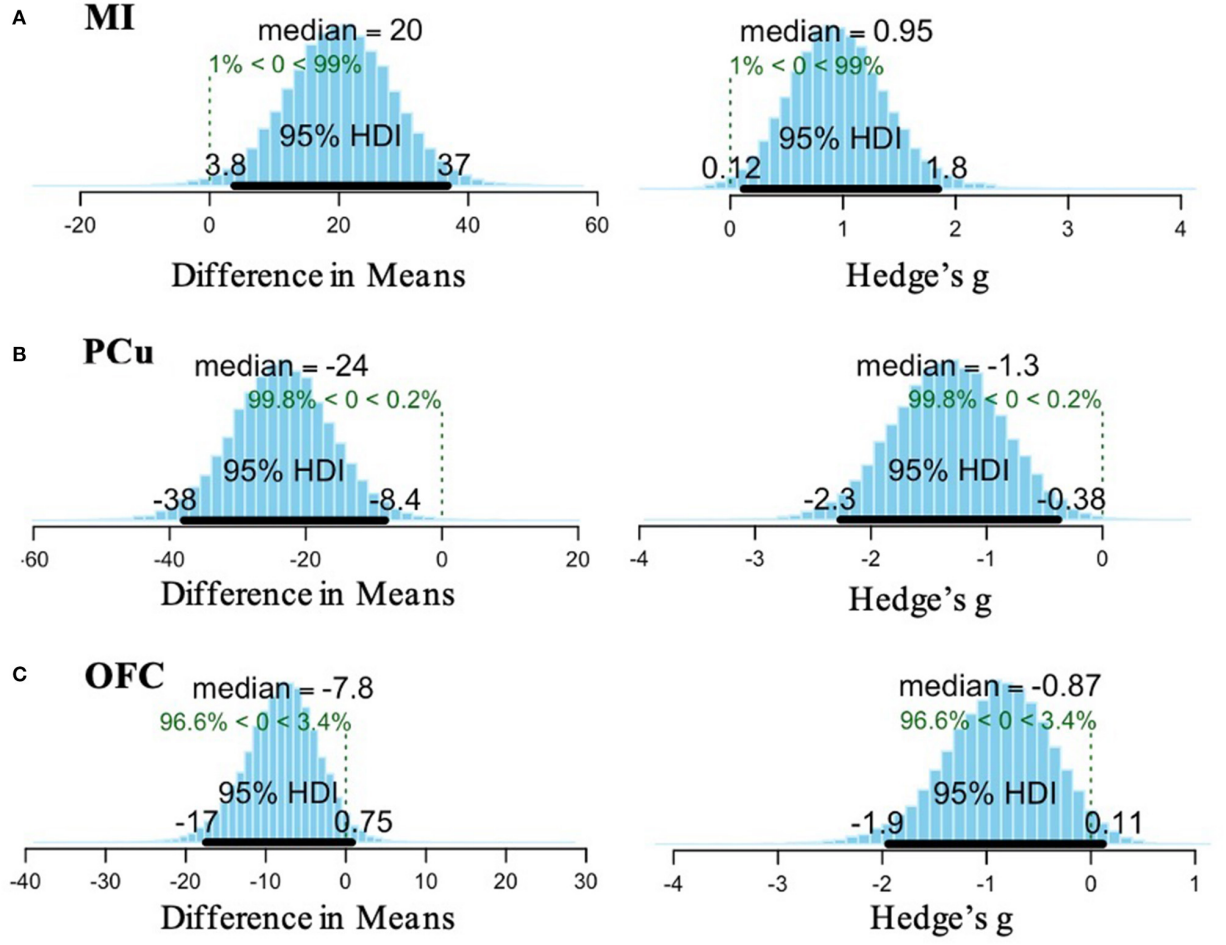
Bayesian analysis results for **(A)** MI, **(B)** PCu, and **(C)** OFC. **(A)** Posterior distribution for the difference in means of MI activation between OCD and TDC indicates a 99% probability of the observed effect (left). The effect size (computed as Hedge's *g*) of the difference in means is 0.95 and the 95% high density interval (HDI) indicates the range of 95% of the most credible values for the effect size is 0.12–1.8 (right). **(B)** Posterior distribution for the difference in means of PCu activation between OCD and TDC indicates a 99.8% probability of the observed effect (left). The effect size is −1.3 and the 95% HDI indicates that 95% of the most credible values for the effect size fall between −2.3 and −0.38 (right). **(C)** Posterior distribution for the difference in means of OFC activation between OCD and TDC indicates a 96.6% probability of the observed effect (left). The effect size is −0.87 and the 95% HDI crosses zero [−1.9 to 0.11], so there is less confidence in the stability of this result (right).

A Bayesian *t-*test revealed that the mean difference in MI amplitude found between OCD and TDC was reliably different from 0, based on a posterior distribution of 99% (i.e., a high degree of certainty that the observed effect is different from zero). The median effect size was 0.95, and the 95% HDI indicated that 95% of the most credible values for the effect size fall between 0.12 and 1.8. For the PCu, a posterior distribution of 99.8% was found, suggesting a high probability that the result found is reliably different from 0. The median effect size was −1.3, 95% HDI [−2.3, −0.38]. Within the OFC, 96.6% of the posterior distribution was in favor of the observed difference in means. Although the effect size for this result was large (−0.87), the 95% HDI included zero [−1.9, 0.11], indicating lower credibility of the OFC result (Figure 3).

#### Time-Frequency Responses (MI, PCu, and OFC)

Group average TFR plots were generated and examined qualitatively by selecting for virtual sensors. In the Go condition, the TFR plots for OCD in MI showed weak, ongoing theta oscillations throughout the trial and less ERS of alpha oscillations following button-press compared to TDC. In contrast, TFR plots for this region in the TDC group showed strong, ongoing theta oscillations for the entirety of the trial, and greater ERS of alpha oscillations following button-press (Supplementary Figure 5). In the PCu for the No-Go condition, TFR plots for OCD showed ERS of delta and theta oscillations at the time of stop errors to the No-Go visual cue, along with transient alpha oscillations. In contrast, the TDC showed ongoing delta oscillations prior to and until after the onset of No-Go visual cue, with strong, transient theta oscillations, and weak, transient alpha oscillations following successful stopping after presentation of the No-Go visual cue (Supplementary Figure 6). In the OFC for the No-Go condition, TFR plots for the OCD group showed continuous delta and theta oscillations throughout the trial and strong ERS in alpha oscillations following a stop error. In comparison, TFR plots for the TDC group showed consistent delta oscillation throughout the trial as well as transient ERS of beta oscillations and ERD of alpha oscillations following a stop error (Supplementary Figure 7).

#### Correlations Between Psychometric Scores and Amplitude of Regional Activation in MI and OFC

There was a small-to-moderate, non-significant, negative association between OFC and total CY-BOCS score (t = −1.86, df = 13, *p* = 0.09, r = −0.46). No association was found between amplitude of MI activation and total CY-BOCS scores (t = −0.08, df = 17, *p* = 0.94, r = −0.02) or between amplitude of activation of PCu and total CY-BOCS scores (t = 1.20, df = 18, *p* = 0.25, r = 0.27).

## Discussion

To our knowledge, this is one of the first studies to use MEG in children and youth with OCD and the only study measuring neural response during cognitive task performance in a medication-naïve sample. We did not find significant differences in Go/No-Go task performance in our medication-naïve OCD sample compared to TDC. This is consistent with previous literature indicating that response inhibition performance on lab-based measures appears to be intact in children and youth with OCD (12, 14, 23, 52). Despite intact behavioral performance, the amplitude of neural response while engaging in the Go/No-Go task differed in OCD vs. TDC participants. *Post-hoc* Bayesian analyses that were used to assess the probability of the observed effect indicated a large effect size for between-group mean differences found in MI (following button presses to Go cues) and in the PCu (following successful stopping to No-Go cues). In contrast, Bayesian analyses indicated that between-group differences in the OFC following stop errors were not reliably different from zero. Therefore, contrary to our hypotheses, the present study did not find clear evidence implicating CSTC group-mean differences during response inhibition in a medication naïve group of children and youth with OCD.

Higher amplitude of activation was observed in the MI region in OCD vs. TDC following button presses to the Go cue during the Go condition. Although not a core region of the CSTC, altered MI response in OCD is intriguing given the close connection between MI and the SMA, a key CSTC circuit region that has been implicated in OCD (53). A recent meta-analysis of the fMRI literature found that both adults and youth with OCD showed hyperactivation of motor regions such as the SMA and pre-SMA, measured in tasks of response-inhibition, or task-switching (11). Increased excitability within MI, specifically, has also been shown in adults with OCD (54), including in a recent study that found increased MI excitability during Go/No-Go Task performance in adult OCD that was associated with an earlier (childhood) onset of OCD symptoms (55). Although the MI finding in the present study was not linked to clinical symptoms, a prior study suggested that increased activation in motor cortex in children and adults with OCD may be linked to increased difficulty in inhibiting responses and potentially related to compulsive behavior (56). Importantly, MI has been a successful neural target for low-frequency (inhibitory) brain stimulation in clinical trials aiming to treat OCD symptoms in adults that have not responded to medication or behavioral treatments (57). Time-frequency analysis of MI activation revealed a qualitative pattern of decreased alpha band synchronization (i.e., a lesser increase in the frequency of oscillations) following button-press to Go visual cue in OCD compared to TDC. A prior Go/No-Go MEG study found alpha oscillations increased in a non-clinical sample following either Go or No-Go cues, suggesting that increased alpha oscillations may signal successful attentional modulation to the cue to facilitate either a motor response or motor inhibition (58, 59). The increased MI amplitude and decreased alpha oscillatory activity found here in OCD may indicate suboptimal attentional modulation within this region during a simple visuomotor task (i.e., to Go cue).

While both OCD and TDC groups engaged the PCu following successful stopping to No-Go cue presentation, the OCD group showed significantly lower amplitude of PCu activation and weaker delta and theta band synchronization compared to TDC. The PCu, along with the posterior cingulate cortex, forms a major subdivision of the default-mode network (DMN) (60). The PCu is highly connected to cortical and subcortical structures and thought to be involved in a wide variety of higher-order cognitive tasks, including attention shifting between object features and self-referential processing (61). Altered DMN activation and/or connectivity at rest or during task performance has been implicated across a number of prior studies in OCD (62, 63), including in children and youth (24), and in unmedicated adults with OCD (62, 64, 65). One recent study in unmedicated adults with OCD found reduced connectivity of the PCu in OCD compared to controls at rest that was able to distinguish OCD from control participants with reasonable accuracy (65). The lower amplitude of PCu activation and weaker delta and theta band oscillations found in the present study may represent sub-optimal attention-shifting in OCD.

Prior studies have implicated alterations within key frontal CSTC regions (i.e., within the ACC, IFG and/or OFC) in OCD during response inhibition (11, 14, 66). For example, a prior MEG study showed increased OFC amplitude during a working memory task in adults with OCD compared to controls (67). A recent EEG study showed right IFG hyperactivity during response inhibition performance in a mostly unmedicated pediatric OCD sample compared to controls (14). Both the ACC and OFC were active following stop errors during the No-Go condition across OCD and TDC participants in the current study. Although OFC amplitude was lower in the OCD group compared to controls, our Bayesian analysis indicated that the effect within the OFC was not consistently different from zero. A small to moderate sized negative correlation between the OFC and the total CY-BOCS score was found but was non-significant, potentially due to power limitations in the small sample examined here. A prior study found increased OFC activation in youth with OCD was associated with CYBOCS score improvement following treatment with SSRIs or cognitive behavioral therapy (22). Therefore, evidence from the prior literature suggests that OFC alterations may be present across pediatric and adult OCD during task performance of response inhibition, though the direction of findings may not be consistent, and may be influenced by age and/or medication exposure.

A major strength of the current study is that investigation of a medication-naïve sample ensured that reported results are not driven or confounded by the effects of pharmacotherapeutic intervention on brain response (26). However, due to the challenge of recruiting medication-naïve participants at a tertiary care centre resulting in the small sample examined here, interpretation of our results must take into account the potential for inflated false positives in a small exploratory example. To mitigate concerns regarding the credibility of our results in our small sample, we used a Bayesian framework to provide additional information regarding the probability of the observed effects. These analyses indicated a high probability that group-mean differences found within the MI and PCu were stable and reliably different from zero with large effect sizes. Other limits include the minimal characterization of the TDC sample and the wide age-range among study participants as MEG-measured neural response may be influenced by age-related differences in brain maturation affecting both processing speed and neural response (68).

### Conclusion

In conclusion, we used MEG to measure neural activity during response inhibition performance in a medication-naïve sample of children and youth with OCD. Our study findings add to prior evidence of intact response inhibition in pediatric OCD. Contrary to our hypotheses, we did not find altered frontal CSTC engagement during response inhibition in our medication-naïve sample. In contrast, alterations in neural response localized to regions outside of the CSTC circuitry that have been implicated in OCD in prior studies. As most prior research in pediatric OCD includes a mix of medicated and unmedicated participants, our findings suggest that additional imaging research in medication-naïve samples is needed to clarify regional differences associated with OCD vs. influenced by medication effects, and suggest that MEG may be sensitive to detecting such differences. Future experiments should aim to recruit larger samples of children and youth with OCD to replicate findings, relate MEG-measured neural response to relevant behavioral and symptom domains, and examine the effects of intervention.

## Data Availability Statement

The datasets presented in this article are not readily available because we do not have participant consent to release this data publicly. Requests to access the datasets should be directed to Stephanie Ameis, stephanie.ameis@utoronto.ca.

## Ethics Statement

The studies involving human participants were reviewed and approved by Hospital for Sick Children Research Ethics Board. Written informed consent to participate in this study was provided by the participants' legal guardian/next of kin.

## Author Contributions

EN completed all data processing and analysis, wrote the manuscript, and made the figures. CD assisted with data processing. JA assisted with statistical analyses. CD, AW, TT, JA, SM, DM, PA, and SA edited the manuscript. All authors contributed to the article and approved the submitted version.

## Conflict of Interest

The authors declare that the research was conducted in the absence of any commercial or financial relationships that could be construed as a potential conflict of interest.
