# Supplementary material for: Visuomotor Activation of Inhibition-Processing in Pediatric Obsessive Compulsive Disorder: A Magnetoencephalography Study
Source: Front Psychiatry. 2021 Apr 29;12:632736. doi: 10.3389/fpsyt.2021.632736 (PMC8116532; doi:10.3389/fpsyt.2021.632736)
Supplement: Supplementary file 1 [file Data_Sheet_1.DOCX]

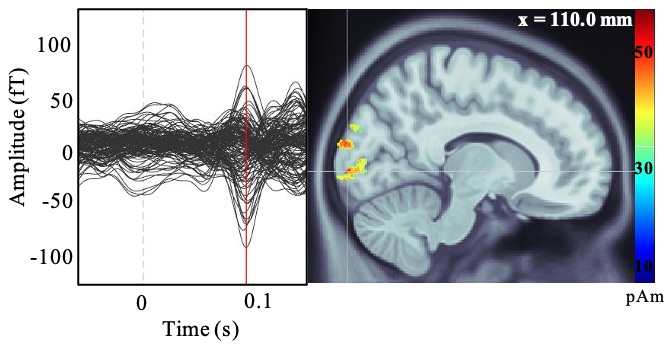


**Supplementary Figure 1.** Group average latency (left) measured in seconds, and source localization (right) measured in pAm, of visual (V1) response in the control group to visual cue in the Go condition.

**
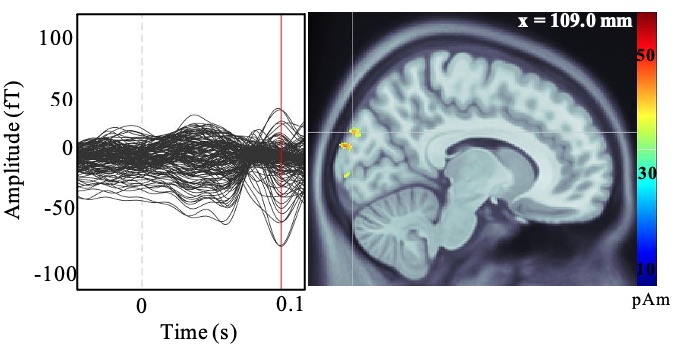
**

**Supplementary Figure 2.** Group average latency (left) measured in seconds (s), and source localization (right) measured in picoamperes (pAm), of visual (V1) response in the OCD group to visual cue in the Go condition.


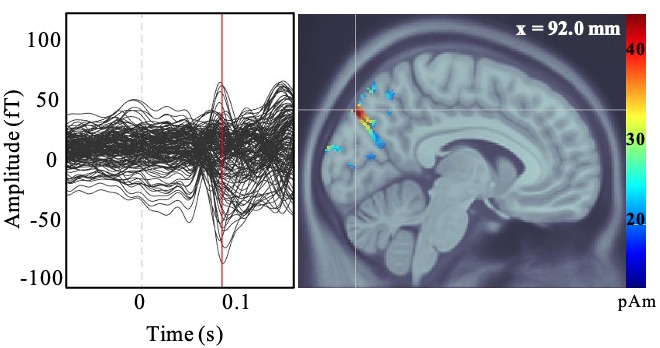


**Supplementary Figure 3.** Average group latency (left) measured in seconds (s), with red line depicting time of peak activation. Average group source localization (right), measured in picoamperes (pAm), of V1 and precuneus during visual response of control group in No-Go condition.

**
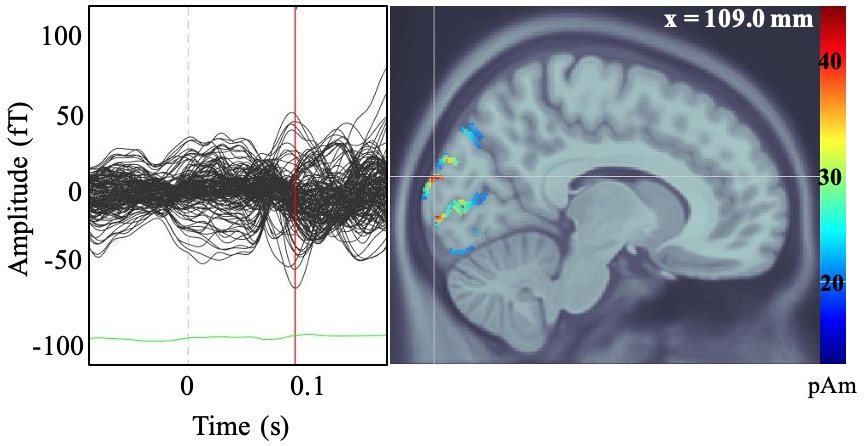
**

**Supplementary Figure 4.** Average group latency (left), measured in seconds (s), and average group source localization (right), measured in picoamperes (pAm), of V1 and precuneus during visual response of OCD group in No-Go condition. The green line represents the global field power (GFP).

**
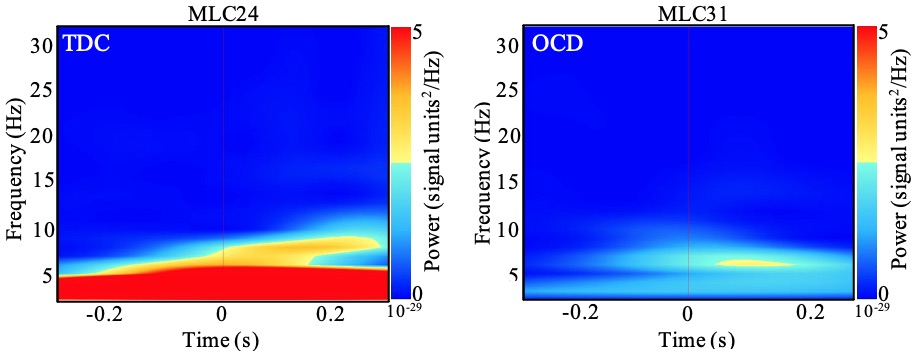
**

**Supplementary Figure 5**. Average group time-frequency response of motor (MI) region activation following Go visual cue in TDC group (left) and OCD group (right). TDC showed strong, ongoing theta oscillations for the entirety of the trial, and greater ERS of alpha oscillations following button-press (left). OCD showed weak, ongoing theta oscillations throughout the trial and less ERS of alpha oscillations following button-press compared to TDC (right).


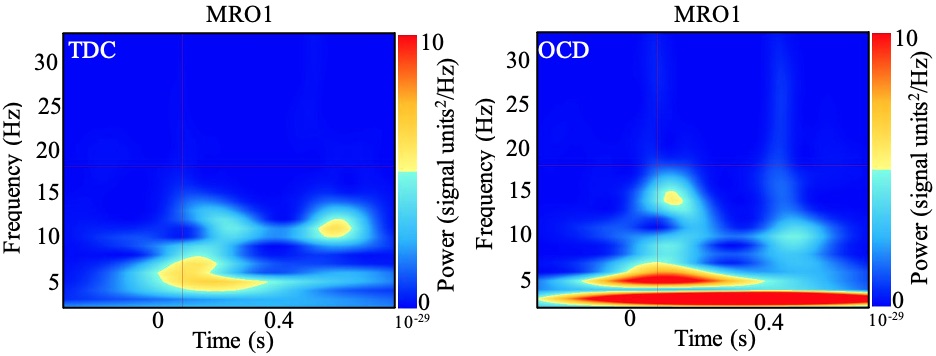


**Supplementary Figure 6**. Average group time-frequency response of precuneus activation following successful stopping to No-Go cue visual cue in TDC group (left) and OCD group (right). TDC showed ongoing delta oscillations prior to and until after the onset of No-Go visual cue (0 ms), with strong, transient theta oscillations, and weak, transient alpha oscillations following successful (left). OCD showed ERS of delta and theta oscillations following successful stopping, along with transient alpha oscillations (right).


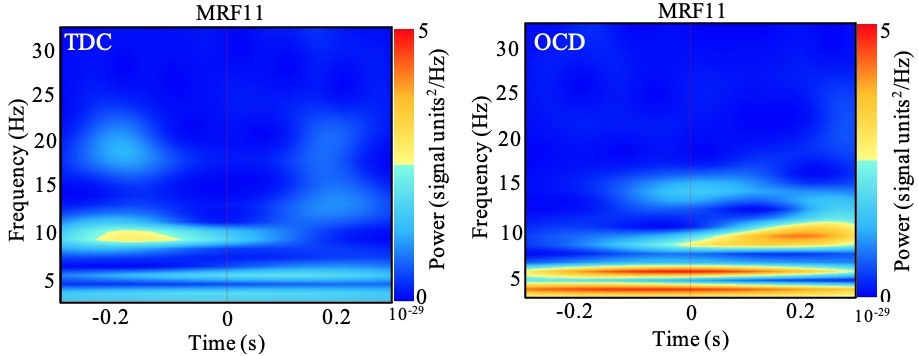


**Supplementary Figure 7**. Average group time-frequency response of orbitofrontal cortex activation following button-press response to No-Go visual cue in TDC group (left) and OCD group (right). TDC showed consistent delta oscillation throughout the trial as well as transient ERS of beta oscillations and ERD of alpha oscillations following a stop error (left). OCD showed continuous delta and theta oscillations throughout the trial and strong ERS in alpha oscillations following a stop error (right).
